# Neutrophil gelatinase-associated lipocalin/lipocalin2, derived from gut crypt cells, exerts intestinal antimicrobial effect via bacterial stimulation of Toll-like receptor 4 and 9

**DOI:** 10.1186/cc11952

**Published:** 2013-03-19

**Authors:** K Mori, T Igarashi, K Inoue, T Suzuki, H Morisaki, J Takeda

**Affiliations:** 1 Keio University School of Medicine, Tokyo, Japan

## Introduction

Neutrophil gelatinase-associated lipocalin (NGAL)/ lipocalin2, known as a sensitive biomarker of acute kidney injury, prevents bacterial iron uptake, resulting in the inhibition of its overgrowth [1]. We previously demonstrated that this protein was discharged into gut lumen from crypt cells in septic conditions, and inhibited the growth of *Escherichia coli *[2]. However, it remains unclear which pathway is associated with the upregulation of NGAL. We therefore designed the present study to reveal whether the pattern-recognition receptor of bacteria, the Toll-like receptor (TLR) family, plays a pivotal role for NGAL secretion from gut crypt cells.

## Methods

With our institutional approval, the ileum and colon of male C57BL/6J mice (6 to 7 weeks) were everted and washed by Ca^2+ ^and Mg^2+ ^free PBS buffer five times. Tissues were incubated with Ca^2+ ^and Mg^2+ ^free PBS containing 30 mM EDTA for 1 hour to isolate crypt cells of gut. The cell suspension was filtered through a cell strainer (40 μm) twice, and deposited the crypt cells by centrifugation at 700×*g*. The isolated crypt cells were resuspended in PBS and stained with 0.25% amido black for labeling paneth cells. The 5×10^5 ^crypt cells were resuspended in 50 ml HBSS containing 2.5% fetal bovine serum and 1% penicillin-streptomycin. The crypt cells were incubated at 37°C with or without TLR ligands: lipopolysaccharide (TLR4 ligand, 10 μg/ml) and CpG-DNA (TLR9 ligand, 8 μg/ml). After a 2-hour incubation period, the crypt cells were deposited and eluted mRNA to measure the expression of both NGAL and TLR mRNA using real-time PCR.

## Results

More than 70 to 80% of collected cells were stained by amido black. LPS significantly upregulated the expression of NGAL and TLR4 mRNA in ileum and colon crypt cells (*P <*0.05). Although the CpG-DNA did not upregulate NGAL and TLR9 mRNA in ileum crypt cells, the apparent expression of NGAL and TLR9 mRNA was found in colon crypt cells (*P <*0.05).

## Conclusion

Bacterial stimulation of TLR4 and TLR9 pathways plays a pivotal role in the expression of NGAL mRNA in gut, suggesting that NGAL, derived from gut crypt cells, could contribute to the regulation of the intraluminal microfiora in the critically ill.
